# Determination of IL-6 Gene Promoter Polymorphism in Patients with Hepatitis C and Its Impact on RNA Secondary Structure

**DOI:** 10.3390/medicina60030368

**Published:** 2024-02-22

**Authors:** Sarah Sadiq, Mohammad Zeeshan Anwar, Huma Shafique, Syed Mohsin Manzoor, Shaiza Shoaib, Rabia Hamid, Shoaib Naiyer Hashmi, Naeem Mahmood Ashraf, Tayyaba Afsar, Mashooq Ahmad Bhat, Suhail Razak

**Affiliations:** 1 Combined Military Hospital, Khariyan Medical College, Gujrat 49600, Pakistan; sarahsadiq10@gmail.com (S.S.); snhashmihistopathologist@yahoo.com (S.N.H.); 2 Institute of Cellular Medicine, Newcastle University Medical School, Newcastle University, Newcastle upon Tyne NE1 7RU, UK; 3 School of Biochemistry & Biotechnology, University of the Punjab, Lahore 54590, Pakistan; 4 Department of Community Health Sciences, College of Applied Medical Sciences, King Saud University, Riyadh 11451, Saudi Arabia; 5 Department of Pharmaceutical Chemistry, College of Pharmacy, King Saud University, Riyadh 11451, Saudi Arabia

**Keywords:** promoter polymorphism, interleukin-6, HCV

## Abstract

*Background and Objectives*: A polymorphism in the promoter region of the IL-6 gene would influence the level of IL-6 expression in patients with HCV, resulting in a pro-inflammatory response. Few studies have shown the association between −174G>C (rs1800795) and −1363G>T (rs2069827) polymorphisms and HCV infection, and their results have been contradictory. There are no data published in our population to study such an IL-6 stimulus against HCV infection and its impact on RNA secondary structure. Therefore, we isolated human subjects from the province of Punjab, Pakistan. The objective was to screen for IL-6 gene promoter polymorphisms −174G/C and −1363G/T and those correlated with serum concentrations of IL-6 in patients with HCV and compared with a control. *Materials and Methods*: In conventional PCR, measurement of serum IL-6 by CLIA and statistical analysis were performed to observe the genotype association studies. By integrating bioinformatics and computational tools, our study aimed to provide a comprehensive understanding of how variations in the promoter region of IL-6 may have functional implications on gene expression. *Results*: The −174G>C and −1363G>T genotypes in the promoter region of patients with HCV were in strong allelic association (Δ = 0.97, *p* < 0.001). Interestingly, the bioinformatics analysis was well aligned with our experimental data. *Conclusions*: Based on the data, it can be inferred that IL-6 gene promoter polymorphisms are important in the dysregulation of IL-6 levels in patients with HCV.

## 1. Introduction

Hepatitis C virus (HCV) is a significant public health concern. Around 185 million people are affected every year worldwide [1]. The hepatitis C virus (HCV) is a single-stranded RNA virus of the Flaviviridae family [2]. Cytokines mainly play a role in immune cell differentiation, maturation, and functional activation. They affect the resistance against hepatitis viruses and therefore the immune response [3]. The single-nucleotide polymorphisms of the interleukin genes could affect the pathogenesis and treatment of patients with HCV. The various other cellular processes such as cell proliferation and differentiation could affect the product of antiviral treatment in the acute phase response and pro-inflammatory and anti-inflammatory pathways [4].

The human IL-6 gene encompasses five exons and four introns, encoded on chromosome 7p21. Numerous single-nucleotide polymorphisms (SNPs), with high statistical significance, have been reported in the IL-6 gene [5]. Certain polymorphisms influence interleukin-6 levels [6]. The IL-6 production levels have been controlled precisely at the transcriptional and translational levels [7]. The IL-6 gene polymorphisms are designated to consist of regulation at the transcriptional stage, e.g., the (−174G/C) polymorphism [8], the (−597G/A) polymorphism, and the (−572G/C) polymorphism. The −1363G/T has been rarely understood in viral-induced chronic infection. Only limited data have been published, which are known to affect the serum concentration of IL-6 [8,9].

According to a literature review, the IL-6 SNPs have been described to progress in the histology development and clinical consequences of HCV but with discrepancy in the correlation with hepatitis-related HCC [9,10]. According to an estimation, seropositive anti-HCV antibodies have increased from 2.3% to 3.8%, as covered in 2019 in the Pakistani population [10]. With the increase in age, IL-6 values increased by 0.05 pg/mL (95% CI: 0.02, 0.09; *p* < 0.01) [10,11].

The serum IL-6 levels have been suggested as biomarkers for a poor prognosis of patients with HCV. The IL-6 promoter SNPs may be a probable risk factor that contributes to the vulnerability to chronic liver diseases [12]. The continuous dysregulation in the synthesis of IL-6 due to a polymorphism slowly activates the downstream immune response, along with oxidative stress, to aggravate inflammation and ultimately be the leading cause of chronic liver diseases [13].

The studies have shown a significant association between the (−174G/C) rs1800795 polymorphism and HCV infection [14]. On the other hand, (−572C/G) rs1800796, and (−596G/A) rs1800797, have been reported to be related to a wide range of diseases, including cancer, autoimmune diseases, and hepatitis [15]. Limited clinical data are available regarding the relationship between IL-6 (−174G>C) (rs1800795) and (−1363G>T) (rs2069827) promoter polymorphisms and chronic HCV infection [16,17,18]. Therefore, our study aimed to investigate the levels of IL-6 and find the tendency to chronic HCV infection association with an IL-6 promoter polymorphism in an isolated subject. The IL-6 polymorphisms could cause disease by affecting crucial RNA–protein interactions. It is still under study that by changing a protein-binding motif, protein binding can be altered, and such changes can affect RNA secondary structure. In our study, it was detected that single-nucleotide polymorphisms can affect RNA–protein interactions from outside binding motifs through an altered RNA secondary structure.

The current study screened for IL-6 gene promoter polymorphisms −174G/C and −1363G/T and those correlated with serum concentrations of IL-6 in patients with HCV and compared them with a control in the Pakistani population.

## 2. Methods

### 2.1. Study Design and Location

The study design was a prospective cohort design. Samples were taken from CMH, Khariyan, Punjab. Clinical and demographic variables were examined. The complete blood count, liver, renal function tests, bilirubin levels, and alpha-fetoprotein (AFP) were tested in all participants. Informed written consent was obtained from all patients and was approved by the ethical committee of CMH, Khariyan Medical College.

### 2.2. Sampling Technique

The non-probability convenience sampling technique was applied. A sample of 200 consecutive Pakistani adults, 100 males, and 100 females, who had chronic HCV confirmed by blood tests, and 100 healthy blood donors were used as controls.

### 2.3. Inclusion Criteria

Patients with confirmed HCV infection with interferon therapy were included.

### 2.4. Exclusion Criteria

Patients with certain cancers such as hepatocellular carcinoma and those with cardiovascular diseases were excluded.

### 2.5. DNA Extraction

Genomic DNA extraction from blood was carried out with organic solvents (5). The DNA was concentrated (Vacuum Concentrator 5301, Berlin, Germany) for 2 or 3 min and dissolved in 200 μL of Tris–EDTA (TE) buffer.

### 2.6. Conventional PCR Method

Two single-nucleotide polymorphisms of the IL-6 gene, in the promoter region −1363G>T (rs2069827), and −174G>C (rs1800795), 1500 upstream of the TSS Promotor region were selected from the whole genome sequence of the IL-6 gene. The primers used are shown in Table 1 and Table 2. Primers were optimized for annealing temperature through the gradient PCR method. The reaction mixture used and conditions for PCR are as below.

#### 2.6.1. Cycling Conditions: SNP-1363

Holding stage95 °C- 5 min

Denaturation95 °C- 1 min

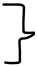


Annealing62 °C- 45 s
**35**
Extension72 °C- 1 min
Final Extension72 °C- 10 min

Hold4 °C
**~**




#### 2.6.2. Cycling Conditions: SNP-174

Holding stage95 °C- 5 min

Denaturation95 °C- 1 min

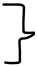


Annealing57 °C- 45 s
**35**
Extension72 °C- 1 min
Final Extension72 °C- 10 min



Purified PCR products were run on 1.5% agarose gel. An amount of 30 mL of 1.5% gel was prepared by adding 0.45 g of agarose in 1 × TBE buffer. The solution was then heated for 1 min in a microwave and cooled down slightly before adding 5 µL ethidium bromide. Then, 5 µL of PCR purified samples were loaded on the gel after adding 1 µL of loading dye to it.

### 2.7. Measurement of Serum IL-6 by (CLIA)

Total concentrations of IL-6 in serum samples were measured using a commercial CLIA kit according to the manufacturer’s instructions (IL-6 kit Elecsys 100 test, Pervaiz, ROCHE, Lahore, Pakistan). The intensity of the developed colour was measured by reading the optical absorbance at 450 nm using a microplate reader. Results were expressed as a pictogram of cytokine per millilitre plasma (pg/mL).

### 2.8. Statistical Analysis

Data were analysed using IBM SPSS software package version 28.0. Quantitative data are described using range (minimum and maximum), mean, standard deviation, and median. The significance of the obtained result was observed at the 5% level. Comparisons between groups were performed by chi-square test for categorical variables to compare between the different groups, and Student’s *t*-test for normally quantitative variables to compare between two studied groups. A χ^2^ test was used to compare the observed numbers of each genotype with those expected for a population in Hardy–Weinberg equilibrium. A value of *p* < 0.05 was considered to be statistically significant.

### 2.9. Bioinformatics Analysis

To examine the functional implications of the −174G/C and −1363G/T variations in the promoter region of IL-6, on the expression of IL-6 mRNA, a comprehensive computational approach was utilized.

### 2.10. Prediction of RNA Secondary Structure

To determine the effect of the aforementioned, promoter variants on the secondary structure of IL-6 mRNA, the RNAfold webserver [19] was utilized. The secondary structure predictions of the wild-type and variant sequences were compared to assess any potential functional consequences.

### 2.11. Annotation of Regulatory Elements

RegulomeDB [20] was utilized to annotate the variants in the IL-6 promoter region and their potential impact on the binding of transcription factors. This database integrates various sources of data to evaluate the potential disruption of regulatory regions by SNPs. Therefore, it contributes to our understanding of the functional impacts of SNPs.

### 2.12. Prediction of Transcription Factor Binding Sites

The AliBaba 2.1 software as used by Li et al., 2021 was employed to predict the potential binding sites of transcription factors within the IL-6 promoter region [21]. Subsequently, we assessed the effects of the −174G/C and −1363G/T variants on these predicted binding sites. Computational models were generated to determine whether these variants alter the affinity of transcription factors, thereby influencing the expression of IL-6.

## 3. Results

We began our study by organizing the data into two main types: qualitative (descriptive) data and quantitative (numerical) data. For the descriptive data, we used numbers and percentages to summarize our findings. To see if there were any notable differences, we used a statistical test called the chi-square test. For the numerical data that followed a normal distribution (the usual bell curve), we calculated the average (mean) and the standard deviation (SD) to understand the spread and used the Student’s *t*-test to compare groups. For data that did not follow this normal distribution, we found the median (the middle value) and range and used the Mann–Whitney test for comparison. We considered our findings significant if there was less than a 5% chance (*p* ≤ 0.05) that they could have occurred by random chance.

**Figure 1:**
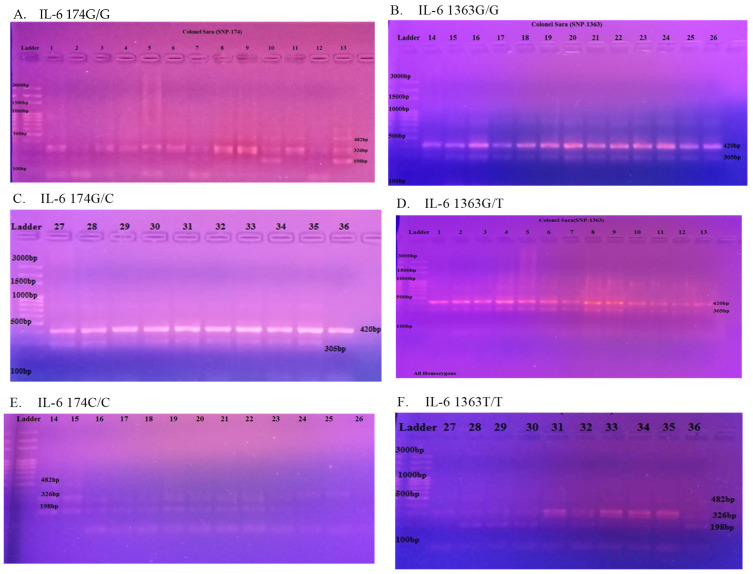
Agarose gel electrophoresis (2%) of PCR products and genotyping of the IL-6 174 and 1363 SNPs. Lane 1, 100 bp marker ladder; (**A**) SNP 1L-6 174 (G/G). All are homozygous, lanes = 420 bp band samples. (**B**) IL-6 1363 G/G; genotype; lanes = 420 bp band samples. (**C**) IL-6 174 G/C; Lanes = 420 bp band samples. (**D**) IL-6 1363 G/T; lanes 1, 2, 5, 7, 10, 12, 13 = heterozygous; 326 bp band samples. (**E**) IL-6 174C/C; lanes = 326 bp band samples. (**F**) IL-6 1363 T/T; 326 bp band samples.

One of the highlights of our research is shown in Figure 1. We used a technique called agarose gel electrophoresis to visualize the PCR products related to the genotyping of IL-6 174 and 1363 SNPs, the two genetic variations we were studying. In Figure 1, gel C, band 27 until band 36 of IL-6 −174G>C, demonstrated the most significant association towards the HCV infection at 420 bp.

We found a strong genetic link between the −174G>C and −1363G>T genotypes, indicating these genetic variations often occur together and are significant in the context of HCV (hepatitis C virus) infection (Table 3).

The allele frequencies of both SNPs in the isolated subjects were 0.37 (IL-6 −174G>C) and 0.07 (IL-6 −1363G>T (Table 3). Genotype distributions were in Hardy–Weinberg equilibrium (*p* > 0.05). IL-6 −174G>C were found to be linked together and to show high allele frequency as compared to IL-6 −1363G>T. G alleles; both SNPs are commonly found in the Pakistani population. On the other hand, the frequency of the C and T alleles of IL-6 −174 and −1363 was reported at a much lower (0.07) frequency (Table 3).

We also investigated the frequency of these IL-6 polymorphisms (genetic variations) among patients with HCV compared to healthy individuals. We observed notable differences in these frequencies, suggesting a potential role of these variations in the susceptibility to HCV.

We also compared various demographic and clinical characteristics, such as age, gender, and several blood test markers, between people with different IL-6 genotypes and healthy controls. One standout finding was the significant difference in age groups, particularly among middle-aged females who showed elevated clinical parameters, pointing to a potential risk factor or marker for HCV infection severity (Table 4).

A key finding was the significant increase in IL-6 levels in patients with HCV compared to healthy controls. This suggests that the presence of HCV infection is associated with higher IL-6 levels, a marker of inflammation, which correlates with the severity of the disease.

Our genetic analysis revealed that the distribution of genotypes for the two IL-6 polymorphisms was as expected as per Hardy–Weinberg equilibrium (Table 5). The comparison of genotype frequencies between patients with HCV and controls showed significant differences, especially for certain genotypes. This reinforces our idea that these genetic variations are linked to HCV infection.

**Figure 2:**
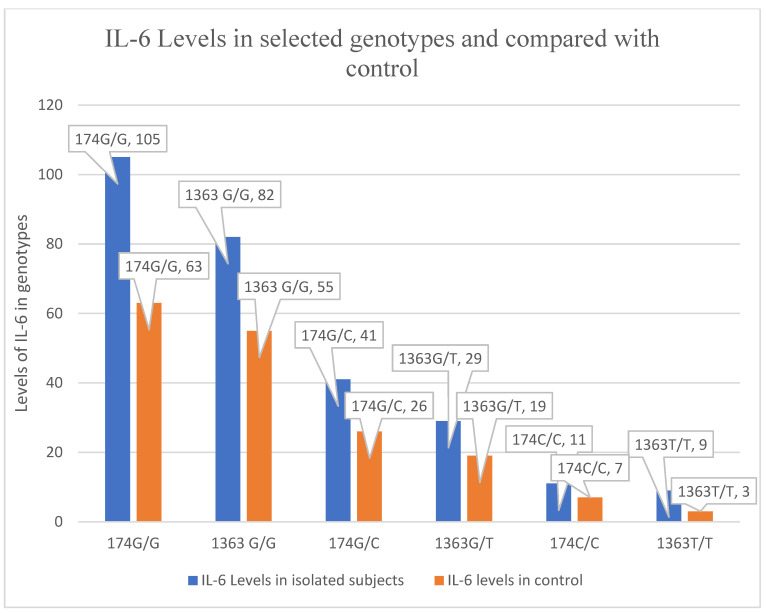
The mean levels with standard deviation of serum IL-6 174G/G, G/C, C/C and 1363G/G, G/T, T/T genotypes were compared in hepatitis C positive patients and control. On pairwise comparison by chi-square test among the polymorphisms, it depicted that the mean IL-6 levels are significantly raised in 174GG, and 1363GT in HCV-positive patients as compared to 174GG and 1363GT in the control.

An interesting observation was that individuals with certain combinations of genotypes (−174GC and −1363G/T) had higher levels of IL-6. This suggests that these genetic variations might influence how the body responds to inflammation. It may also be inferred that certain genotypes possibly offer better control in the inflammatory response (Figure 2).

**Figure 3:**
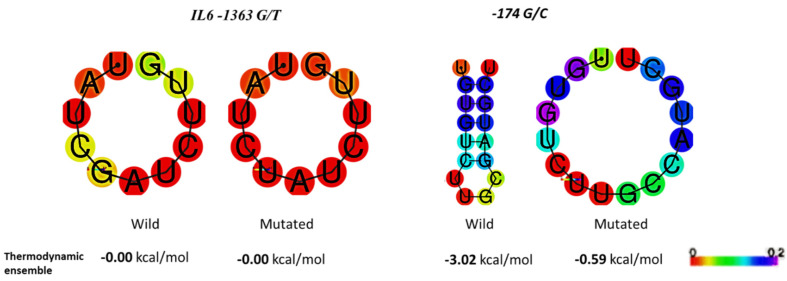
IL-6 promoter region variations impact its mRNA secondary structure and positional entropy. Different colours represent varying amounts of positional entropy. Red represents low entropy, while blue represents high entropy. The gradient of hues shows intermediate levels of entropy, from red to orange, yellow, parrot green, green, cyan, and blue.

We also explored how these genetic variations impact the structure of mRNA (Figure 3). The −174G/C mutation caused a noticeable change in the RNA structure. This suggests that this genetic variation could affect how genes are expressed and how proteins are made. The −1363G/T variation seemed to have less effect on mRNA structure as compared to the −174G/C.

**Figure 4:**
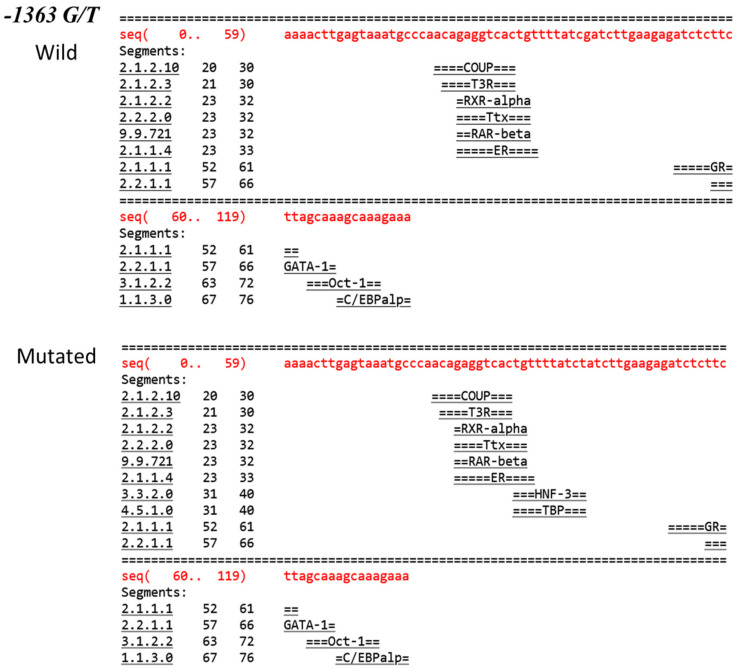
Influence of IL-6 -1363 G/T variation on its transcription factor binding sites. The analysis covers two segments of a DNA sequence: positions 0 to 59 and 60 to 119. The tool identifies potential binding sites for various transcription factors such as COUP, T3R, RXR-alpha, and others, within these segments. The symbols “===“demarcate the length and location of each predicted binding site along the DNA sequence. The data suggest a complex regulatory potential, with multiple transcription factors likely interacting with the sequence, which may have implications for the gene’s expression and regulation in the cellular context.

**Figure 5:**
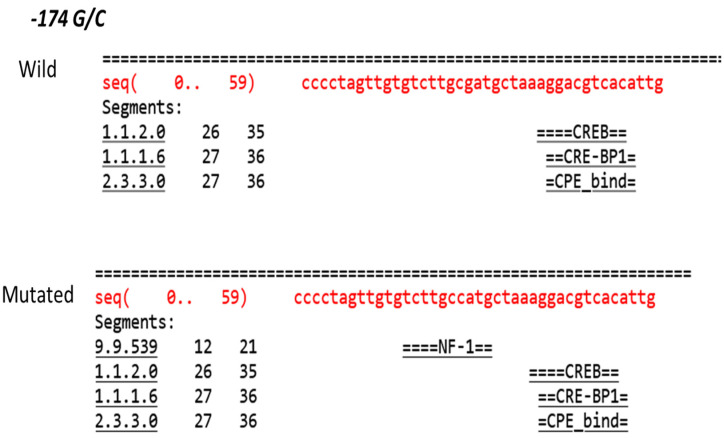
Influence of IL6 −174 G/C variation on its transcription factor binding sites, The image represents transcription factor binding sites (TFBS) within a single DNA sequence segment spanning from nucleotide positions 0 to 59. The analysis identifies specific binding sites for transcription factors such as CREB, CRE-BP1, CPE_bind, and NF-1, indicated by their respective position ranges. The red text represents the DNA sequence under investigation, while the black text shows the transcription factors’ names alongside the corresponding sequence segments where their binding is predicted.

The computational analysis also revealed that these variants might change the expression of the IL-6 gene. This happens through altering the binding sites for transcription factors on the IL-6 gene (Figure 4 and Figure 5). Such changes could importantly affect the body’s reaction to infections or inflammation (Table 6).

In summary, our detailed exploration uncovered significant links between genetic variations in IL-6 and HCV infection. These findings suggest that these genetic markers could influence not only the risk of HCV infection but also how the body responds to such infections. This in-depth examination into the genetic and molecular underpinnings of HCV infection opens-up new avenues for understanding the disease and potentially for guiding treatment and prevention strategies.

## 4. Discussion

The study described the effect of genetic polymorphisms found in the IL-6 gene promoter region results on the pro-inflammatory effect of IL-6 levels in patients with hepatitis C. The statistical data found for the polymorphism suggested that IL-6 genotypes in the promoter region may be a significant contributing factor to virus-induced chronic infection and can be compared with the results of Cussigh A et al. in 2011 [4]. Their work focused on clarifying the role of IL-6 haplotypes in facilitating progressive disease in patients with chronic HCV infection. The −174G/C and −1363G/T variants at the IL-6 gene were systematically selected according to the previous study by Hamed et al. in 2018 in different populations. The statistical significance of the −174GC genotype (*p* < 0.0011) and −1363(G/T), *p* = 0.0052 showed a trend toward higher IL-6 levels, in direct contrast to the initial published data [18].

The significant association between the −174C allele and increased IL-6 levels has recently been reported in a sample of patients with virus-induced chronic infection [16]. The IL-6 −174 genotype appears to affect not only the physiological response but also the more lethargic response related to virus-induced chronic infection. Such explanations might dominate the potential problem of comparing the important in vitro and in vivo evaluations of functionality [13].

Interestingly, subjects of genotype −174GC and −1363G/T appeared to raise IL-6 levels compared to C and T allele carriers, reflecting a more controlled response to the inflammatory stimulus. The absolute concentration of IL-6 levels was significantly different from genotype −174CC and −1363T/T. The genotype might influence the active profile of the IL-6 response as compared to the control (Figure 2). However, more detailed investigations are required in other clinical representations along with more thorough in vitro studies.

Our data are well aligned with recent findings, thereby providing a more profound comprehension of the influence of RNA structure mutations on gene expression. The research conducted by Lin and Zhang (2023) on Venezuelan equine encephalitis virus reveals that mutations in coding regions of non-structural proteins can exert substantial control over gene expression [22]. These data are analogous to our observations regarding the impact of the −1363G/T and −174G/C variants on IL-6 expression. This regulatory phenomenon implies a convoluted mechanism in which specific mutations have the potential to disrupt the structural integrity of RNA. Therefore, it may have the potential to affect patterns of gene expression.

Moreover, Hurst and Chen (2021) demonstrated the SNP ability to induce significant alterations in the stability of RNA [23]. This finding aligns with our data. Our data suggest that −174G/C mutation increases positional entropy. Therefore, it suggests a destabilization of mRNA structure. It may impede the translation process.

The broader implications of mutations on gene expression, as demonstrated by Xu (2023), offer a contextual framework [24]. This may help us to comprehend the regulatory dynamics influenced by the −1363G/T and −174G/C variants. It may be inferred that these variations not only impact RNA structure but also alter the regulatory mechanisms. Both things may change the structure and biological function of the protein.

Cheng et al. (2017) introduced a new way to understand how changes in RNA can affect its structure after mutations [25]. This is crucial for determining if these mutations help to maintain or alter the RNA secondary structure. Similarly, a computer-based study by Sumantri et al. (2020) on changes within the beta-globin gene also supports our findings. This method shows how using computers can help predict what happens when certain changes occur in genes [26].

The details that we identified in this study showed genetic variations add scientific rationality to the results as reported. Probably, the statistical data reported may depict the efficient variants of the IL-6 promoter region. According to the study, different haplotypes might affect the transcriptional regulation, making them a possible influential parameter to investigate further, but haplotypes require a large sample size to statistically impact the multiple comparisons. Moreover, the present study was intended to analyse a single polymorphic variant that is presently being used as a potential biomarker in association studies by clinical research institutes.

The identification of polymorphism of the gene encoding the proinflammatory IL-6 may demonstrate the importance of clinical use. Our results will support the interpretation of data from association studies of IL-6 genotype and virus-induced chronic infection as well as the benefit of exploring novel therapeutic therapies.

On the other hand, our results might suggest that these IL-6 SNPs have a greater influence on an acute IL-6 response to patients with HCV than the IL-6 levels determined by more chronic inflammation. Validation is required in studies in lower grades of inflammatory response and chronic inflammation. These data set the context for exploring the role of IL-6 genotypes in other inflammatory situations, such as in hepatocellular carcinoma.

## 5. Conclusions

In conclusion, we demonstrated for the first time in our studied groups that the IL-6 −174 promoter polymorphisms influence the IL-6 response to the inflammatory stimulus to an extent that is possible to be clinically applicable. Our data supported the fact that IL-6 gene promoter polymorphisms play an essential role in dysregulating the IL-6 levels in patients with HCV. More thorough investigations will be required to discover the probable contribution of IL-6 promoter polymorphisms to HCC progression in HCV-infected individuals, keeping in view the future application of SNPs as biomarkers for onset and prediction of the prognosis of viral-induced chronic infection, as well as to explore better treatment options.

## 6. Limitations of the Study

The polymorphisms studied are functional with in vivo effects. So, the present study requires more data to observe the effects of genetic haplotype by obtaining a very large sample size from other provinces of Pakistan.

## Figures and Tables

**Table 1:** Primer pairs for SNP −1363 to order.

Outer forward 1	CGCGGCAGAGGACCACCGTCT
Outer reverse 1	TGAATCTGCTTCCGCGTCGGCA
Wild allele forward 1	CAACAGAGGTCACTGTTTTATCG
Mutant allele reverse 1	AAGAAGAGATCTCTTCAAGATA

**Table 2:** Primer pairs for SNP −174 to order.

Outer forward primer 2	GAGGAAACTCAGTTCAGAA
Outer reverse primer 2	ATGAGCCTCAGACATCTCCAGTC
Wild allele forward primer 2	TTTCCCCCTAGTTGTGTCTTGCC
Mutant allele reverse primer 2	ATGTGACGTCCTTTAGCATC

**Table 3:** IL-6 Promoter genotype distributions and allele frequency.

Locus	SNP Site	Genotype Distribution			Frequency	*p* Value ≤ 0.05
IL-6 −174	−174	GG	GC	CC	0.37 (0.31–0.43)	GG = 0.005 GC = 0.001 CC = 0.62
		47 (36.5)	76 (63)	13 (10)		
IL-6 −1363	−1363	GG	GT	TT	0.07 (0.04–0.10)	GG = 0.012 GT = 0.0052 TT = 0.765
		31 (27)	45 (32)	3 (1.9)		

**Table 4:** Comparison of clinical and demographic variables between studied groups.

Parameters	Studied Groups		*p* Value ≤ 0.05
	IL-6 −174(GG)	IL-6 −1363(GG)	
Age	46.72 ± (11.01)	51.87 ± (13.01)	<0.001 **
Male (middle-aged)	67 ± (65.5)	38 ± (34.07)	0.734
Female (middle-aged)	31 ± (29.5)	27 ± (23.98)	0.005 *
TLC	6.37 ± (3.20)	6.55 ± (2.99)	0.011 *
Haemoglobin	14.07 ± (2.30)	13.29 ± (3.45)	0.519
ALT	54.0 ± (11.0)	43.0 ± (12.76)	<0.002 *
AST	56.20 ± (11.0)	61.11 ± (11.2)	<0.005 *
AFP	2.90 ± (1.30)	2.41 ± (1.00)	<0.001 *
Alb	4.34 ± (0.70)	4.00 ± (0.89)	0.001 *

** Highly Significant, * Significant.

**Table 5:** Comparison of clinical and demographic variables observed in healthy groups.

Parameters	Controls		*p* Value ≤ 0.05
	IL-6 −174(GG)	IL-6 −1363(GG)	
Age	29.84 ± (7.9)	23.87 ± (9.01)	0.561
Male (middle-aged)	47 (45.3)	37 ± (18.07)	0.698
Female (middle-aged)	51 (49.7)	41 ± (28.67)	0.311
TLC	5.60 (4.90–11.10)	7.81 ± (3.75)	0.217
Haemoglobin	12.18 ± 0.88	9.01 ± (4.87)	0.487
ALT	21.0 (09.0–35.0)	18.04 ± (11.01)	0.435
AST	24.0 (11.0–38.0)	21.00 ± (17.03)	0.837
AFP	3.0 (0.94–8.5)	2.05 ± (2.01)	0.771
Alb	5.10 ± 0.29	3.99 ± (2.07)	0.583

**Table 6:** Prediction of IL-6 −174 G/C and −1363 G/T variation impact on IL-6 expression.

Rs IDs	Rank	Score	Rank Interpretation
rs2069827	1f	0.70823	eQTL/caQTL + TF binding/chromatin accessibility peak
rs1800795	1f	0.55436	eQTL/caQTL + TF binding/chromatin accessibility peak

## Data Availability

Raw data will be available from the corresponding author on request.
